# Time-dependent microbiology of peripancreatic drainage fluid in severe acute pancreatitis: a prospective real-world observational study using metagenomic sequencing and culture

**DOI:** 10.3389/fmed.2026.1795250

**Published:** 2026-06-05

**Authors:** Yinshan Wu, Yifan Gao, Zhendong Fang, Weipeng Huang, Feng Guo

**Affiliations:** Department of Critical Care Medicine, Sir Run Run Shaw Hospital, Zhejiang University School of Medicine, Hangzhou, China

**Keywords:** acute pancreatitis, infected pancreatic necrosis, metagenomic next-generation sequencing, microbiological profiling, percutaneous catheter drainage, time-dependent analysis

## Abstract

**Objective:**

The microbiological characteristics of peripancreatic collections in severe acute pancreatitis (SAP) evolve over time, yet prospective data linking pathogen detection to disease timing and first intervention remain limited.

**Methods:**

This prospective single-center observational study enrolled 20 patients with SAP undergoing first-time percutaneous catheter drainage (PCD) for suspected infected pancreatic necrosis (IPN). Peripancreatic drainage fluid samples were simultaneously analyzed by conventional microbiological culture and metagenomic next-generation sequencing (mNGS). Microbiological positivity rates were compared according to time from disease onset (≤14 vs. >14 days).

**Results:**

Overall, mNGS was positive in 9/20 cases (45.0%) and conventional culture in 6/20 cases (30.0%). When stratified by time from disease onset, microbiological positivity was low within 14 days (mNGS: 1/7, 14.3%; culture: 1/7, 14.3%), but increased in patients undergoing drainage beyond 14 days (mNGS: 8/13, 61.5%; culture: 5/13, 38.5%). mNGS identified a broader spectrum of pathogens, particularly polymicrobial, anaerobic, and fungal organisms. *Enterococcus species* and *Klebsiella pneumoniae* were the most frequently detected pathogens.

**Conclusion:**

In this prospective observational cohort, peripancreatic collections were predominantly culture- and mNGS-negative during the early phase of SAP, supporting the concept that early necrosis is commonly sterile. In later stages, mNGS provides complementary microbiological information beyond conventional culture. These findings offer descriptive real-world evidence on the time-dependent microbiology of suspected IPN and may inform future studies on optimized diagnostic and antimicrobial strategies.

## Introduction

Severe acute pancreatitis (SAP) is a life-threatening form of acute pancreatitis, posing a significant burden on healthcare systems (1). In patients with persistent organ failure, mortality rates can be as high as 20–50% (2). Infectious complications and multiple organ dysfunction syndrome (MODS) account for the majority of deaths in SAP, particularly when infected pancreatic necrosis (IPN) develops (3, 4). While sterile necrosis is associated with relatively lower mortality, the occurrence of IPN markedly increases the risk of death, especially when accompanied by organ failure (5, 6). Therefore, accurate recognition of IPN and decision-making are important considerations in the management of SAP.

In current practice, IPN diagnosis largely relies on a combination of contrast-enhanced computed tomography (CT), microbiological culture, and clinical signs of sepsis (7). However, these traditional methods exhibit significant limitations. Although the presence of intralesional gas on CT is highly specific for infection, imaging findings are often inconclusive, particularly in the early phase of disease (7, 8). Conventional culture of peripancreatic fluid is limited by prolonged turnaround times and reduced detection of fastidious, anaerobic, or fungal organisms, challenges that are further compounded by frequent antibiotic exposure before or during sampling (7, 9, 10). Furthermore, fine-needle aspiration (FNA), once considered a valuable diagnostic tool, is no longer routinely recommended due to its high false-negative rates and invasiveness (8, 11). Despite these diagnostic challenges, the ‘step-up approach’ has become the standard strategy for managing IPN, favoring minimally invasive drainage as the initial intervention and reserving necrosectomy for refractory cases (12, 13). Recent randomized trials further indicate that delayed intervention may allow a proportion of patients to recover without invasive drainage, highlighting the need for careful interpretation of microbiological findings in relation to disease timing and clinical context (14).

Metagenomic next-generation sequencing (mNGS) has emerged as a promising culture-independent tool for pathogen detection in infectious diseases, enabling broad, hypothesis-free identification of bacteria, fungi, and viruses in a single assay (15). Preliminary studies in acute necrotizing pancreatitis and related conditions suggest that mNGS may detect pathogens missed by culture and better characterize polymicrobial and anaerobic infections (7). In addition, mNGS offers a shorter turnaround time and is less affected by prior antibiotic exposure, which is particularly relevant in critically ill patients who often receive empirical antimicrobial therapy (7, 8). However, most existing studies evaluating mNGS in infected pancreatic necrosis are retrospective in nature, frequently include patients with prior abdominal interventions, or rely on fine-needle aspiration samples that may not reflect initial drainage events (7, 11, 16). Consequently, the microbiological characteristics of peripancreatic drainage fluid in patients with severe acute pancreatitis undergoing first-time percutaneous catheter drainage, and their time-dependent relationship with disease course, remain incompletely described.

Therefore, this prospective observational study aimed to characterize the microbiological profile of peripancreatic drainage fluid in SAP patients undergoing first-time PCD for suspected IPN using both mNGS and conventional culture, and to explore its time-dependent relationship with disease course. We hypothesized that microbiological positivity would increase with disease duration and that mNGS would provide broader pathogen profiling than culture, particularly for polymicrobial, anaerobic, and fungal organisms.

## Methods

### Study design and setting

This was a prospective, single-center observational study conducted in the Department of Critical Care Medicine at Sir Run Run Shaw Hospital, Zhejiang University School of Medicine (Hangzhou, China). Adult patients with SAP who underwent first-time PCD between April 2020 and February 2022 were consecutively screened for eligibility. Decisions regarding the indication and timing of PCD, as well as antimicrobial therapy, were made exclusively by the treating clinical teams as part of routine care and were independent of the study investigators. Once a patient with SAP was scheduled to undergo first-time PCD, the investigators assessed eligibility and, if eligible, enrolled the patient and arranged simultaneous submission of peripancreatic drainage fluid for conventional culture and metagenomic next-generation sequencing.

### Definitions

Severe acute pancreatitis (SAP) was defined according to the Revised Atlanta Classification (2012) as acute pancreatitis with persistent organ failure lasting longer than 48 h (17).

Definite IPN was defined by the presence of extraluminal gas within a necrotic collection on contrast-enhanced computed tomography (CT) (13).

Suspected IPN was defined necrotizing pancreatitis patients accompanied by persistent sepsis or progressive clinical deterioration despite maximal intensive care support, in the absence of an alternative source of infection (13).

The term peripancreatic drainage fluid in this study refers to fluid obtained during first-time percutaneous catheter drainage of peripancreatic collections, including acute necrotic collection and walled-off necrosis, as defined by the Revised Atlanta Classification (17).

### Participants

#### Eligibility criteria

Patients were eligible if they met at least one of the following criteria:

1. PCD was deemed necessary by the attending physician based on a diagnosis of definite IPN or clinical suspicion of IPN (suspected IPN);or
2. The patient was enrolled in the TIMING trial, in which drainage timing followed a predefined research protocol.

The TIMING trial is a multicenter randomized study comparing early on-demand drainage versus postponed intervention in patients with acute necrotizing pancreatitis complicated by persistent organ failure (18).

Exclusion criteria were:

1. Pregnancy-associated pancreatitis.
2. Chronic pancreatitis or tumor-related pancreatitis.
3. Previous percutaneous, endoscopic, or surgical interventions for pancreatic necrosis at other institutions.
4. Post–cardiopulmonary resuscitation with unrecovered neurological function.
5. Severe comorbidities, including advanced heart failure, active myocardial ischemia, cardiovascular intervention within the previous 60 days, liver cirrhosis, chronic kidney disease with creatinine clearance <40 mL/min, or chronic obstructive pulmonary disease requiring home oxygen therapy.

### Enrollment and sample size

All eligible patients were enrolled before the PCD procedure. A total sample size of 24 patients was planned based on the anticipated number of eligible SAP patients undergoing first-time PCD during the study period, as the primary aim was descriptive and exploratory. During verification of clinical records, four patients with prior percutaneous drainage at outside hospitals were excluded in accordance with predefined exclusion criteria, leaving 20 patients for the final analysis.

### Data collection

Clinical data were prospectively collected using a standardized case report form, including demographics, etiology of pancreatitis, disease severity scores (APACHE II and SOFA), infection-related biomarkers, laboratory parameters, organ support therapies, and clinical course variables. No imputation was performed for missing data. Detailed information on individual antimicrobial exposure within 48–72 h prior to first percutaneous catheter drainage, including agents used and timing relative to disease onset, is provided in Supplementary Table S2.

### Microbiological sampling and mNGS

Peripancreatic drainage fluid was obtained at the time of first PCD, collected in sterile tubes, and immediately transported to the hospital microbiology laboratory. After aliquoting for routine culture according to standard operating procedures, the remaining sample was reserved for mNGS.

### DNA extraction, library preparation, and sequencing

DNA extraction and library preparation were performed using an automated next-generation sequencing library preparation system (Meridian Biotechnology Co., Ltd., Hangzhou, China), following the manufacturer’s validated clinical protocol. Library quality and adapter content were assessed using an Agilent Bioanalyzer 2,100 in combination with quantitative PCR to ensure suitability for sequencing. mNGS was performed at a certified external laboratory (MatriDx Biotechnology, Hangzhou, China) on an Illumina NextSeq 500 platform, using a standardized clinical pipeline for sequence generation and bioinformatic processing.

### Interpretation of mNGS results

Metagenomic sequencing results were interpreted using a predefined clinical pipeline. Negative controls were included in each sequencing batch to monitor potential background contamination. Host-derived reads were removed during bioinformatic preprocessing according to the standard clinical workflow of the sequencing provider. A microorganism was considered a putative pathogen when the reads-per-million (RPM) ratio of the clinical sample to the negative control met the predefined threshold (RPM_sample/RPM_NC ≥ 5) and the organism was deemed clinically compatible with the patient’s presentation. Interpretation of mNGS results was performed by senior intensivists with experience in the management of severe acute pancreatitis, based on standardized laboratory reports, conventional microbiological results, and the overall clinical context. Final interpretations were reached by consensus.

### Statistical analysis methods

All statistical analyses were performed using R, version 4.3.0 (R Foundation for Statistical Computing, Vienna, Austria). Continuous variables were summarized as mean ± standard deviation (SD) or median with interquartile range (IQR), depending on distribution. Categorical variables were presented as counts and percentages.

Descriptive analyses were used to summarize baseline characteristics, disease severity scores, etiologies, laboratory findings, and clinical outcomes. Days from onset and length of hospital stay were expressed as mean ± SD. Group comparisons were conducted using the chi-square test or Fisher’s exact test for categorical variables, as appropriate. A two-sided *p*-value < 0.05 was considered statistically significant. No adjustments were made for multiple comparisons given the exploratory nature of the study. Within each timing stratum (≤14 vs. > 14 days), paired concordance between mNGS and conventional culture was summarized using 2 × 2 tables, and discordant pairs were compared using McNemar’s exact test.

## Study results

### Clinical characteristics

**Figure 1:**
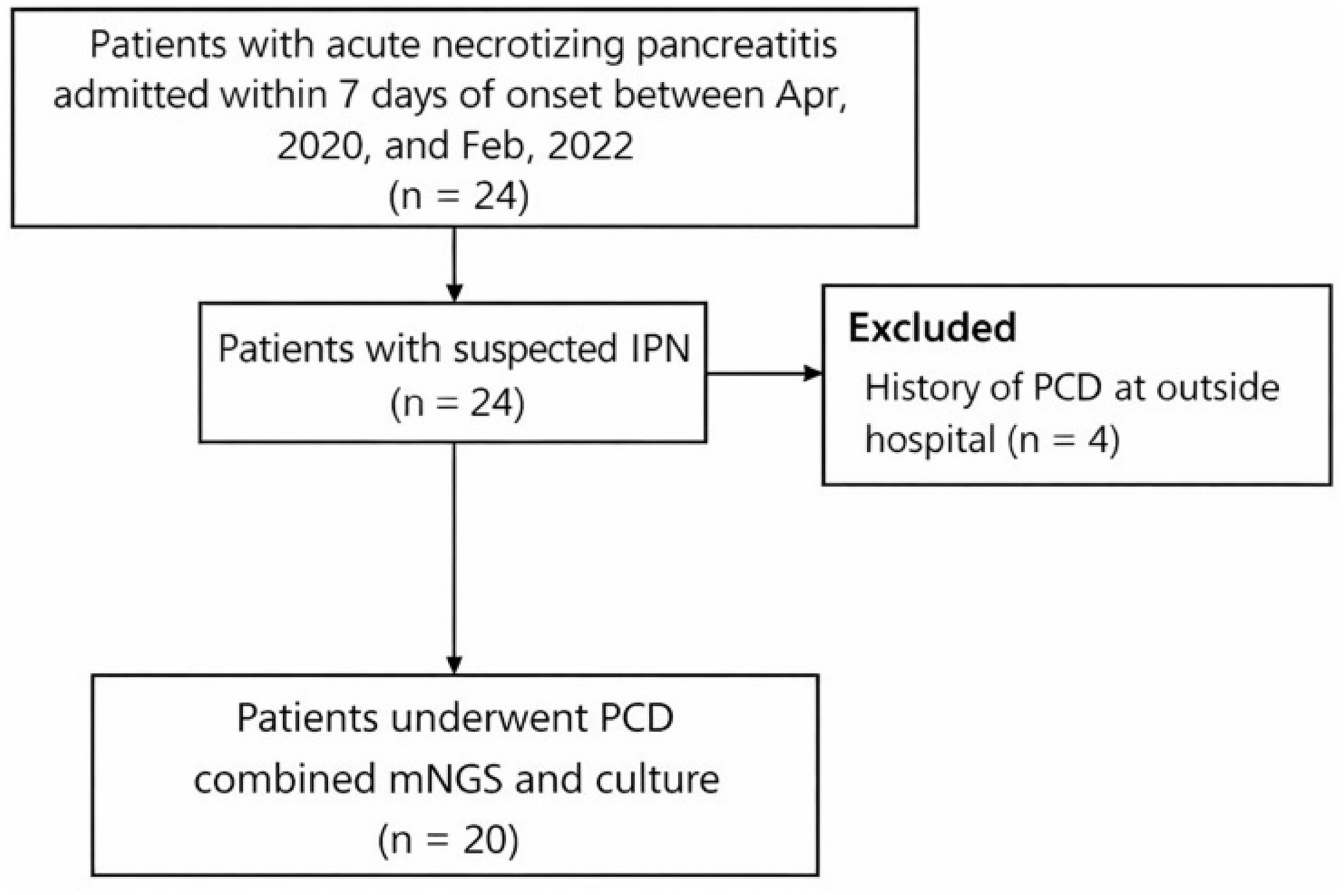
The flow chart of patient selection. IPN, infected pancreatic necrosis; mNGS, metagenomic next-generation sequencing; PCD, percutaneous catheter drainage.

This study enrolled patients from April 25, 2020, to February 1, 2022. A total of 24 patients were initially screened, of whom 4 were subsequently excluded after data verification because of a prior puncture history at outside hospitals, leaving 20 patients for the final analysis (Figure 1). Baseline characteristics are summarized in Table 1, including an overall mNGS positivity of 45.0% (9/20). Individual antimicrobial exposure prior to drainage varied according to clinical context and timing and is summarized in Supplementary Table S2.

**Table 1:** Baseline characteristics of the study population*.

Characteristics	Values (*N* = 20)
Demographic characteristics	
Age (years), median (IQR)	40.50 (32.25, 54.50)
Male sex, number (%)	13 (65.0%)
Clinical characteristics	
Etiology, number (%)	
Hypertriglyceridemia	10 (50.0%)
Biliary	8 (40.0%)
Others	2(10.0%)
Time from onset to drainage (days), median (IQR)	19.50 (13.00, 25.25)
Severity scores	
APACHE II score, median (IQR)	17.00 (13.75, 25.25)
SOFA score, median (IQR)	7.00 (4.00, 9.25)
Laboratory parameters at drainage	
CRP (mg/L), median (IQR)	233.55 (180.80, 278.35)
PCT (μg/L), median (interquartile range)	1.39 (0.94, 5.37)
White blood cell count (×10^9^/L), median (interquartile range)	14.20 (10.93, 21.80)
Neutrophil count (×10^9^/L), median (interquartile range)	12.58 (9.79, 18.93)
mNGS-positive cases, *n* (%)	9 (45.0%)
Clinical outcomes	
Length of hospital stay (days), median (interquartile range)	61.50 (40.25, 99.25)

*Continuous variables are expressed as median (interquartile range, IQR); categorical variables are presented as numbers (percentages). APACHE II, Acute Physiology and Chronic Health Evaluation II; SOFA, Sequential Organ Failure Assessment; mNGS, metagenomic next-generation sequencing; HTG, Hypertriglyceridemia; CRP, C-reactive protein; PCT, Procalcitonin.

### Indications and timing of percutaneous catheter drainage

The indications for percutaneous catheter drainage stratified by timing from disease onset are summarized in Table 2. Among the seven patients who underwent PCD within 14 days, drainage was driven by enrollment in the TIMING trial (*n* = 3) or clinical suspicion of IPN (*n* = 4), with no cases meeting criteria for definite IPN. In contrast, one patient in the >14-day group met criteria for definite IPN based on CT evidence of extraluminal gas.

**Table 2:** Each patient met a single primary indication for PCD.

Primary indication for PCD	Overall (*n* = 20)	≤14 days (*n* = 7)	>14 days (*n* = 13)
Definite IPN	1	0	1
Suspected IPN	16	4	12
TIMING trial enrollment	3	3	0

Definite IPN was defined by the presence of extraluminal gas within a necrotic collection on contrast-enhanced computed tomography (CT); IPN, infected pancreatic necrosis; CT, computed tomography.

### Pathogen spectrum and polymicrobial infections

**Figure 2:**
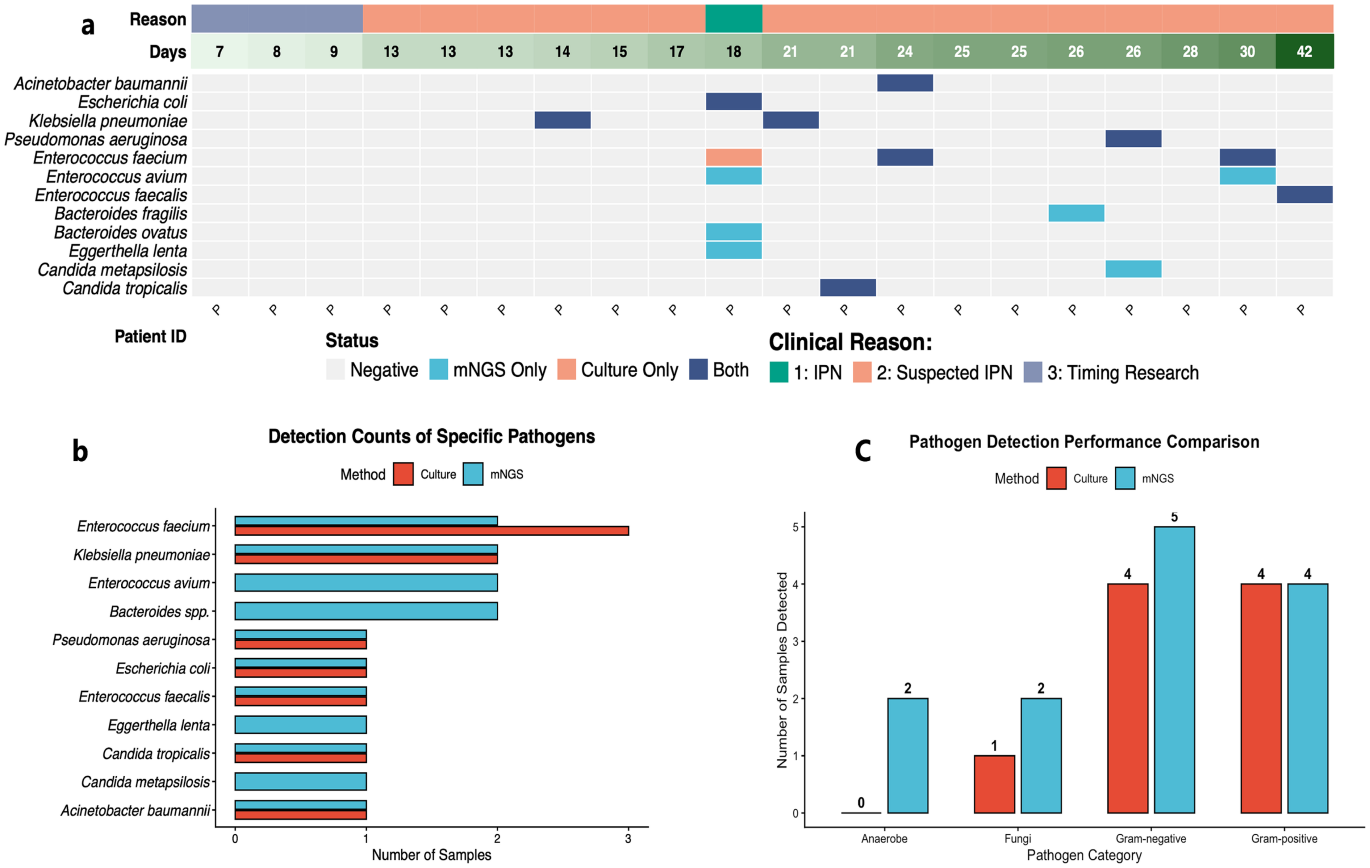
**(a)** Individual-level visualization of drainage timing, clinical indications, and microbiological findings. The top row indicates the primary clinical indication for percutaneous catheter drainage; (1) definite infected pancreatic necrosis (IPN) based on gas detected on contrast-enhanced CT; (2) clinically suspected IPN; (3) enrollment in the TIMING trial. The second row shows the number of days from disease onset to first-time PCD. Rows below depict pathogens detected in peripancreatic drainage fluid by conventional culture and/or metagenomic next-generation sequencing (mNGS). Gray indicates negative results by both methods; blue indicates concordant detection by both methods; light blue indicates mNGS-only detection; and orange indicates culture-only detection. This figure demonstrates the highly selected nature of early drainage cases and the time-dependent increase in microbiological positivity and complexity. **(b)** Pathogens detected by mNGS versus culture. **(c)** Distribution of total detection counts across four major pathogen categories.

The pathogen spectrum of peripancreatic drainage fluid is shown in Figure 2. Gram-negative bacteria predominated, with *Klebsiella pneumoniae* and *Escherichia coli* being the most frequently detected organisms. *Enterococcus species* were also commonly identified. Polymicrobial infections were more frequently observed in the later phase of disease, and mNGS provided more detailed profiling of mixed infections. In a representative case (Patient 15), routine culture identified only two bacterial species, whereas mNGS simultaneously detected five pathogens, including three anaerobic bacteria and one Gram-positive organism, thereby offering a more comprehensive microbiological description of peripancreatic infection.

### Microbiological positivity of mNGS and culture over time

Among the 20 SAP patients undergoing first-time PCD for suspected IPN, mNGS was positive in 9/20 cases (45.0%), while conventional culture was positive in 6/20 cases (30.0%). When stratified by time from symptom onset to PCD, positivity was low within 14 days (1/7, 14.3% for both mNGS and culture) but increased thereafter, reaching 8/13 (61.5%) for mNGS and 5/13 (38.5%) for culture in patients drained at >14 days (Table 3). Although these temporal differences did not achieve statistical significance (Fisher’s exact test *p* = 0.070 for mNGS and *p* = 0.354 for culture), they suggest a time-dependent increase in microbiological yield from peripancreatic collections, with numerically higher detection by mNGS compared with culture, particularly beyond 14 days after onset. Paired concordance analyses between mNGS and conventional culture showed high agreement overall and within each timing stratum, with discordant results predominantly observed as mNGS-positive/culture-negative pairs, particularly in patients undergoing drainage >14 days from disease onset (Supplementary Table S1). These analyses are descriptive given the small sample size.

**Table 3:** Correlation between timing of percutaneous catheter drainage and microbiological positivity rate*

Time from onset to PCD	mNGS positive cases, n/N (%)	Culture-positive cases, *n*/*N* (%)
≤14 days (*n* = 7)	1/7 (14.3)	1/7 (14.3)
>14 days (*n* = 13)	8/13 (61.5)	5/13 (38.5)
*p*-value (Fisher’s exact test)	0.070	0.354

*Data are presented as *n*/*N* (%). Intergroup comparisons were performed using two-sided Fisher’s exact test. PCD, percutaneous catheter drainage; mNGS, metagenomic next-generation sequencing. These correlations are exploratory and not adjusted for multiple testing.

**Figure 3:**
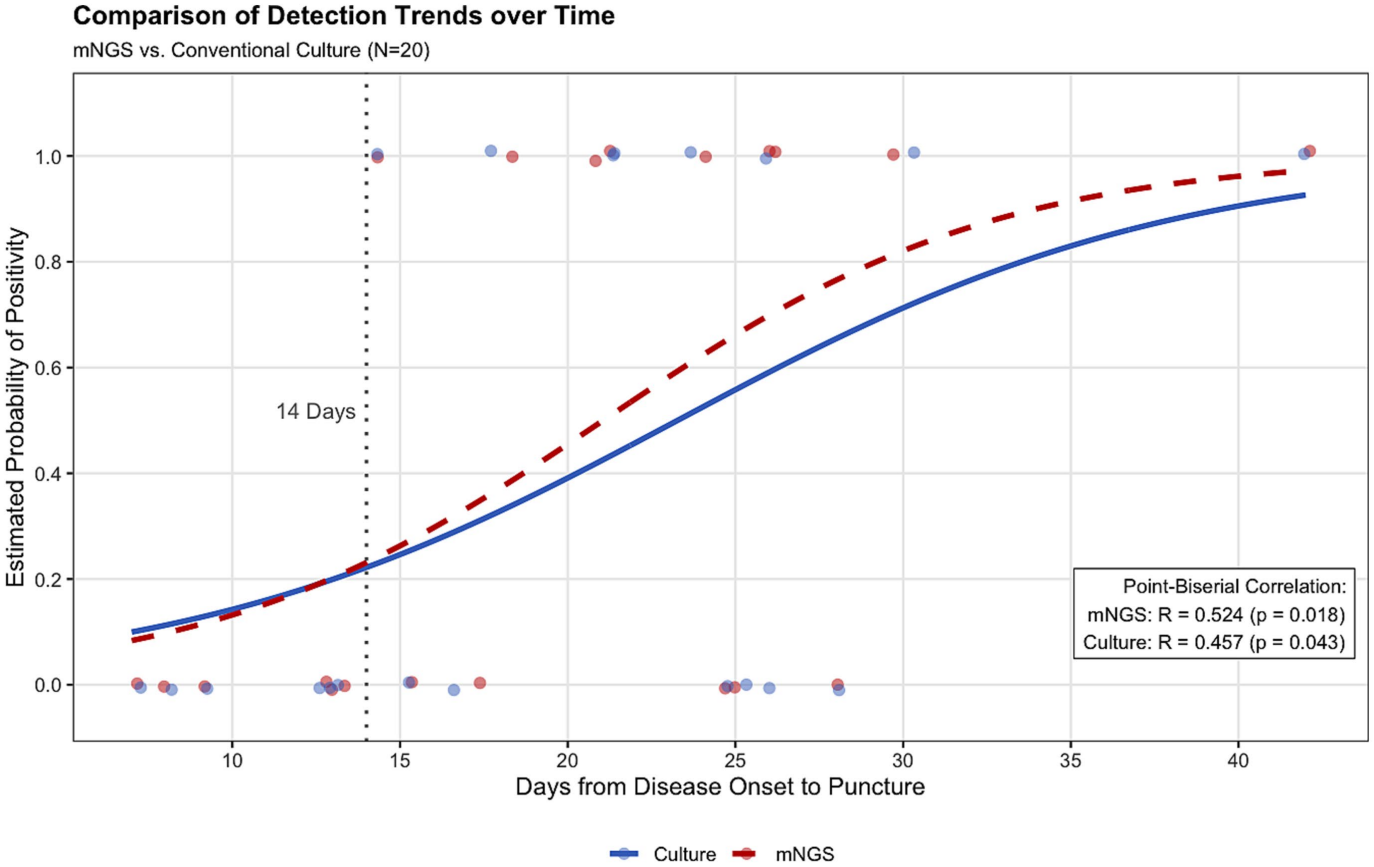
Point-biserial correlation between mNGS positivity and time from disease onset (*R* = 0.524, *p* = 0.018). These analyses are exploratory and hypothesis-generating.

Figure 3 illustrates the increase in positivity rates for both methods over time, with the mNGS curve consistently above the culture curve, suggesting numerically higher detection probability for mNGS across the observed time window.

## Discussion

### Principal findings

In this prospective observational study, we describe a pattern suggesting time-dependent increase in the microbiological findings of peripancreatic drainage fluid in patients with severe acute pancreatitis undergoing first-time percutaneous catheter drainage. Microbiological positivity was uncommon in collections drained within 14 days from disease onset, whereas pathogen detection increased at later time points. Importantly, early drainage cases in this cohort occurred exclusively in highly selected clinical contexts, including trial-related enrollment or strong.

clinical suspicion of infected pancreatic necrosis, rather than routine early intervention.

### Time-dependent microbiological positivity and clinical context

These observations align with the current understanding that pancreatic necrosis is frequently sterile in the early phase of disease and that secondary infection tends to emerge later in the clinical course (17, 19). The low microbiological positivity observed in early-drainage cases should be interpreted in the context of their highly selected nature and does not necessarily reflect routine early management of severe acute pancreatitis (12, 14, 17). Together, these findings emphasize the importance of considering disease timing when interpreting microbiological results from peripancreatic collections.

### Microbiological profile and the complementary role of mNGS

The pathogen spectrum observed in this cohort was dominated by gut-derived organisms (20, 21), including Gram-negative bacteria and *Enterococcus species*, and frequently involved polymicrobial infections. This distribution is consistent with the pathophysiology of necrotizing pancreatitis and prolonged critical illness (4, 22), in which disruption of intestinal barrier function and translocation of enteric organisms may occur. The frequent detection of polymicrobial and anaerobic pathogens highlights the complexity of late-stage peripancreatic infections.

Rather than replacing conventional culture, metagenomic next-generation sequencing provided complementary microbiological information in this real-world cohort. mNGS detected a broader range of organisms, particularly anaerobic and fungal pathogens, and revealed discordant findings in a subset of cases. These features may be particularly relevant in later-stage disease, where prior antimicrobial exposure and polymicrobial infection are common. However, given the descriptive nature of this study, the clinical impact of mNGS-guided antimicrobial adjustment could not be assessed and requires further investigation.

### Implications for antibiotic stewardship and future directions

The very low microbiological positivity of drainage fluid within 14 days strongly supports existing guidelines that discourage routine prophylactic antibiotics for SAP without clear evidence of infection (23–26). Prior studies have shown that prophylactic antibiotic use does not reduce the incidence of IPN or mortality and may contribute to multidrug-resistant organisms and fungal superinfections (25, 26). In our institution, antimicrobial management for severe acute pancreatitis has generally followed current guideline recommendations. Empirical antibiotic therapy, when initiated for suspected infection, primarily targets Gram-negative organisms, and agents with activity against intrinsically resistant *Enterococcus species*, including carbapenem-resistant strains, are typically reserved for later stages or for cases with microbiological or strong clinical justification. Within this context, the present findings provide descriptive microbiological observations that may generate hypotheses relevant to future antimicrobial decision-making studies, particularly in the later phase of disease. The frequent detection of *Enterococcus species*—predominantly *Enterococcus faecium*—as well as polymicrobial and anaerobic organisms in drainage fluid obtained beyond 14 days suggests that pathogen spectra may be broader and more complex than initially anticipated. These observations raise the hypothesis that, in selected late-stage cases with suspected infected pancreatic necrosis, earlier consideration of antimicrobial coverage beyond Gram-negative organisms warrants prospective evaluation in future studies. Whether mNGS findings translated into changes in antimicrobial management or improved clinical outcomes was not assessed in this study.

Nevertheless, given the observational nature of this study, the small sample size, and incomplete quantification of prior antibiotic exposure, such implications for antibiotic stewardship should be interpreted cautiously. Prospective, outcome-oriented studies incorporating standardized antimicrobial strategies and longitudinal microbiological assessment are required to determine whether earlier or broader antimicrobial coverage can translate into improved clinical outcomes. To enhance transparency and aid interpretation of microbiological findings, particularly the observed discordance between mNGS and conventional culture, detailed information on individual antimicrobial exposure prior to first percutaneous catheter drainage is provided in Supplementary Table S2.

### Limitations

This study has several limitations. First, this was a single-center prospective observational study with a relatively small sample size (*n* = 20). As the primary objective was descriptive and exploratory, the sample size was determined by the number of eligible patients undergoing first-time PCD during the study period rather than by a formal power calculation. Accordingly, the study was not designed to detect statistically significant differences between mNGS and conventional culture, and the findings should be interpreted as hypothesis-generating. Second, all clinical management decisions, including the indication and timing of PCD and antimicrobial therapy, were made by the attending physicians according to routine clinical practice rather than being protocolized by the study. While this pragmatic design reflects real-world decision-making and enhances external validity, it may also introduce heterogeneity in patient management and potential selection bias that could not be fully controlled. Third, although individual antimicrobial exposure within 48–72 h prior to the first PCD was fully documented in electronic medical records and is summarized in Supplementary Table S2, these data were not prospectively collected as a primary study variable. Accordingly, the quantitative impact of prior antibiotic exposure on mNGS and culture results could not be formally assessed, given the small sample size and heterogeneity of antimicrobial regimens. In addition, the impact of mNGS findings on antimicrobial modification or clinical outcomes was not formally assessed. Therefore, no causal inferences regarding the effect of antimicrobial strategies or mNGS-guided management can be drawn. Finally, this study focused on the microbiological characteristics of initial peripancreatic drainage fluid only. Further studies incorporating longitudinal sampling, clinical outcomes, and multicenter cohorts are warranted to better define the role of mNGS in the management of SAP with suspected IPN.

## Conclusion

In this prospective single-center observational study of SAP patients undergoing first-time PCD, microbiological positivity of peripancreatic drainage fluid showed a numerical time-dependent increase. Early collections (≤14 days) were frequently microbiologically negative, whereas pathogen detection increased in later stages. mNGS provided broader pathogen profiling compared with conventional culture, particularly for complex infections. Given the exploratory nature and limited sample size of this study, these findings should be interpreted as descriptive and hypothesis-generating, and warrant confirmation in larger, multicenter cohorts.

## Data Availability

The raw data supporting the conclusions of this article will be made available by the authors, without undue reservation.
