# Supplementary material for: Time-dependent microbiology of peripancreatic drainage fluid in severe acute pancreatitis: a prospective real-world observational study using metagenomic sequencing and culture
Source: Front Med (Lausanne). 2026 Jun 5;13:1795250. doi: 10.3389/fmed.2026.1795250 (PMC13279767; doi:10.3389/fmed.2026.1795250)
Supplement: Supplementary file 1 [file Table_1.DOCX]

## Table 4. Paired concordance between mNGS and conventional culture, overall and stratified by timing

A. Overall (n = 20)

|  | Culture + | Culture − | Total |
| --- | --- | --- | --- |
| mNGS + | 8 | 1 | 9 |
| mNGS − | 0 | 11 | 11 |
| Total | 8 | 12 | 20 |
| Concordance | 95.0% |  |  |
| McNemar (exact) | p = 1.00 |  |  |

B. Early drainage (≤14 days, n = 7)

|  | Culture + | Culture − | Total |
| --- | --- | --- | --- |
| mNGS + | 1 | 0 | 1 |
| mNGS − | 0 | 6 | 6 |
| Total | 1 | 6 | 7 |
| Concordance | 100% |  |  |
| McNemar | Not applicable |  |  |

C. Late drainage (>14 days, n = 13)

|  | Culture + | Culture − | Total |
| --- | --- | --- | --- |
| mNGS + | 7 | 1 | 8 |
| mNGS − | 0 | 5 | 5 |
| Total | 7 | 6 | 13 |
| Concordance | 92.3% |  |  |
| McNemar (exact) | p = 1.00 |  |  |

Footnote: Paired concordance between metagenomic next-generation sequencing (mNGS) and conventional culture is shown. Concordance was defined as both tests positive or both negative. McNemar’s exact test was used to compare discordant pairs within each stratum. Given the small sample size, statistical analyses are descriptive and should be interpreted with caution.
