# Supplementary material for: Time-dependent microbiology of peripancreatic drainage fluid in severe acute pancreatitis: a prospective real-world observational study using metagenomic sequencing and culture
Source: Front Med (Lausanne). 2026 Jun 5;13:1795250. doi: 10.3389/fmed.2026.1795250 (PMC13279767; doi:10.3389/fmed.2026.1795250)
Supplement: Supplementary file 2 [file Table_2.DOCX]

| Patient ID | Antimicrobial exposure within 48–72 hours before PCD | Days from disease onset to drainage (days) |
| --- | --- | --- |
| 4 | NA | 7 |
| 2 | NA | 8 |
| 16 | NA | 9 |
| 7 | NA | 13 |
| 11 | Cefoperazone–sulbactam | 13 |
| 21 | NA | 13 |
| 19 | Piperacillin–tazobactam | 14 |
| 24 | Piperacillin–tazobactam & Linezolid | 15 |
| 14 | Biapenem | 17 |
| 15 | Cefoperazone–sulbactam | 18 |
| 12 | Imipenem–cilastatin & Vancomycin | 21 |
| 23 | Piperacillin–tazobactam | 21 |
| 9 | Imipenem–cilastatin & Linezolid | 24 |
| 6 | Tigecycline & Meropenem | 25 |
| 8 | Cefoperazone–sulbactam | 25 |
| 1 | Imipenem–cilastatin | 26 |
| 17 | Cefoperazone–sulbactam | 26 |
| 20 | Cefoperazone–sulbactam | 28 |
| 3 | Cefoperazone–sulbactam | 30 |
| 13 | Tigecycline | 42 |

Supplementary Table S2. Individual antimicrobial exposure prior to first percutaneous catheter drainage*

* PCD, percutaneous catheter drainage. Antimicrobial exposure refers to systemic antibacterial or antifungal agents administered within 48–72 hours prior to the first PCD procedure. Patients are ordered according to the interval between disease onset and drainage. Antimicrobial regimens were determined by the treating physicians based on routine clinical practice rather than a predefined protocol.
